# Topics of Public Interest

**Published:** 1911-01

**Authors:** 


### TOPICS OF PUBLIC INTEREST

### Contagious Diseases Hospital discussed before Joint Committee of Common Council.

**An expert opinion—Secretary of State Charities Association gives his Views—Decries County Plan—Believes that Institution should be Centrally Located in City.**

[From *Buffalo Express* December 14, 1910.]

HOMER FOLKS, secretary of the State Charities Aid Association, outlined the opinions of the standing committee on hospitals of the association which had a bearing on the situation in Buffalo. Mr. Folks explained that the views were necessarily at long range, since the fifteen members of the hospital committee of the association lived in New York.

There were, however, the expressions of men acquainted with hospital work and needs. Their general knowledge had been supplemented by information gleaned here by Bailey B. Burritt, assistant secretary of the association and in charge of the hospital branch, and by Nicholas Hansen, assistant to Mr. Burritt. The association had undertaken the task at the request of the joint committee. Secretary Folks supplemented the report with some views of his own.

The attendance at the session of the joint committee December 13, 1910, was made up of representatives of the Chamber of Commerce and Manufacturers' Club, and of the local hospitals. In general, the statements of Mr. Folks were agreed with and there was not much discussion. The session served to confirm the facts regarding hospitals given to the committee a few weeks ago by the State Board of Charities and by committees from various bodies and physicians and citizens who have interested themselves in the subject.

Briefly, what Mr. Folks had to say made more certain the conviction that if a hospital is to be built to take care of advanced cases of tuberculosis, contagious diseases and other forms of sickness which corporate hospitals cannot care for, then the city and not the county should build the hospital. The site for the hospital should be central to the population to be served. If a plot of from

40 to 50 acres, centrally located, can be procured, then not only would there be no danger from contagion, but there would be no injury to property values, no matter in what section of the city the hospital was located.

Mr. Folks expressed himself in a way not complimentary to the manner in which the board of supervisors managed hospitals. He was not speaking of Erie County in particular. Supervisors are merely legislative bodies, with no administrative heads, and, therefore, fail when they undertake administrative tasks. It is the opposite with cities. There is no law providing for the conduct of county hospitals for contagious diseases. There is a law providing for their administration by cities

A STIGMA ATTACHES.

County hospitals are run as adjuncts to poorhouses. Even if the hospital has a distinct location from the poorhouse, the stigma attaches, since, like the poorhouse, the hospital is from necessity under the management of the poor department of the county. Secretary Folks cited instances of families exhausting all their means before allowing a sick member to be taken to a county hospital. He knew of instances where suicide was committed by those who could not bear the stigma of being pauper patients.

He proved by examples the danger of having contagious diseases hospital too far removed from the population to be served. To take an ill child five or six miles in an ambulance lessened the chances for that child to recover.

"Can you explain why Cleveland and Baltimore built their hospitals on the outskirts?" asked Health Commissioner Fronczak.

Mr. Folks said that he was speaking of the experience of New York and added that the proportion of children who do not recover in contagious hospitals was greater than it should be. Councilman Bingham asked if Mr. Folks was to be understood that the proportion of deaths was greater than would occur at the homes. Mr. Folks replied that he meant that the proportion of deaths was greater than if the children had been treated at the institutions in which they contracted the sickness and he believed the cause was to be found in carrying the children long distances.

He considered the site of the Erie County Hospital too far removed from the center of population. He pictured the new hospital as one conducted within the means of the ordinary citizen and warned the committee that it must plan upon the basis that the people, who are grasping the importance of hospital care, are on the increase. Now only the wealthy and the extremely poor receive the best of modern medical treatment. The same treatment must be brought to the class between, which will not accept charity.

SCHOOL FOR NURSES.

Provision for the treatment of acute and subacute cases, such as the corporate hospitals treat, was recommended for the new hospital to the number of 100 beds. The advantage would be that a school of nurses could be carried on in conjunction with the hospital.

With pupil nurses the Boston City Hospital, with 286 patients, had a nurse bill of $7,000, while the contagious hospital in Buffalo, with only 86 patients had a nurse cost of $11,700. A school for nurses cannot be maintained in an exclusively contagious hospital, and in Buffalo trained nurses have to be employed. By the city taking hold and building the combined hospital, Mr. Folks showed that not only would there be a far less administrative cost by saving duplication in the administrative heads and in the employees, but a saving in the building cost.

If the county should build its planned hospital of 500 beds, 200 beds for chronic, 200 beds for tuberculosis, and 100 beds for acute cases, he estimated the cost at $1,250,000. For the city to build its separate hospital of 200 beds for contagious cases and twenty beds for detention cases, he estimated the cost on the same basis at $550,000, a total of $1, 800,000, while if the city took up the whole task, and built both hospitals, with the one administrative building and other economical features resulting from centralisation, the cost would not exceed $1,514,000, a saving of $264,000.

He gave it as his belief that it was advisable for the city to send to the corporate hospitals all acute cases, since they can be taken care of at a charge of $1 a day. Even with that arrangement he did not consider a provision of 100 beds for acute cases too many for the city hospital.

Councilman Mahoney asked Mr. Folks if he considered the opposition of a locality as sufficient ground for abandoning a projected site for the hospital.

"If you permit opposition to be conclusive you will not locate it anywhere," said Mr. Folks. Still, he explained that he would hesitate about locating the hospital in a residential neighborhood, even if the harm done would be only fanciful, unless there was sufficient land area about the hospital. His experience was that land values increased about a well-kept and beautiful hospital.

Charles W. Pardee, president of the Buffalo General Hospital, had doubts about combining the contagious hospital with the hospital for chronic cases and cases of tuberculosis. He said that in the city of London with seven centuries of hospital experience isolation was strictly enforced. Mr. Folks said that was not true of continental cities. He gave the opinion of Dr. Hermann M. Biggs, general medical officer of New York City department of health, that a contagious hospital should be centrally located and its establishment with a general hospital, even on a site of ten acres to provide for a city like Buffalo, would not have the slightest effect on the health of the residents of the locality nor any bad results for the hospital patients.

Mr. Folks brought up the example of Paris hospitals, where in the same building different classes of diseases were handled. Mr. Pardee replied that the Paris hospitals were the poorest managed in the world. Dr. Fronczak said that was not true of the Pasteur Institute than which there was none better managed and there he saw wards for different kinds of contagion, separated only by glass partitions.

During the discussion the question of congestion at the county hospital last winter came up. Mr. Pardee said repeatedly the poor authorities had been called up and urged to send the patients, suffering from acute and subacute forms of illness to the city hospitals and the authorities took no action. The city hospitals at that time had ample room. He could assign no reason for their course and they offered none. It was made clear that it was the county poor department and not the city department that so acted.

Dr. Walter S. Goodale, superintendent of the contagious diseases hospital, put a line of questions, which brought from Mr. Folks the explanation that he considered the payment of $60 a month to trained nurses at the contagious hospital reasonable. It was difficult to procure pupil nurses in New York just now. The same condition was reported for Buffalo.

Mrs. Bernard Bartow of the Children's Hospital explained that the standard for nurses had been raised in Buffalo. No compensation was now given to pupil nurses and a higher grade of women are entering the service. When a nurse is given her certificate she is paid $100. She expressed her earnest desire that the county almshouse and hospital would be separated. She said that a contagious pavilion had been added to the Children's Hospital in Bryant street. There was slight opposition at first. It has worked well. There has been no depreciation of property on the street.

At the close of the session, the joint committee, consisting of aldermen Staples, Burley, and Weimar, and councilmen Bull, Mahoney, and Dorr went into executive session. It was expected that a report would be submitted the following Monday that the city should build the hospital. The expectation, however, was not fulfilled.

